# Optogenetic activation of nigral inhibitory inputs to motor thalamus in the mouse reveals classic inhibition with little potential for rebound activation

**DOI:** 10.3389/fncel.2014.00036

**Published:** 2014-02-11

**Authors:** Jeremy R. Edgerton, Dieter Jaeger

**Affiliations:** Department of Biology, Emory UniversityAtlanta, GA, USA

**Keywords:** optogenetics, motor thalamus, basal ganglia, bursting, inhibition

## Abstract

The inhibitory output from the internal pallidum and substantia nigra to the thalamus forms an important link in the transmission of basal ganglia processing to cortex. Two hypotheses consider either inhibition of thalamic activity or thalamic excitation via post-inhibitory rebound burst firing as the functional mode of this link. We used optogenetics to characterize the synaptic properties of nigral input to motor thalamus in adult mouse brain slices, and to determine in what conditions the nigral inhibition of motor thalamus is transmitted via inhibition or rebound firing. Our results are more consistent with graded inhibition of spiking for conditions expected in normal awake animals, because inhibitory potentials from nigral input were generally not sufficient to elicit rebound spikes when the thalamic neurons were actively firing. However, with bursty or fast trains of nigral input low-threshold rebound spike bursts could be triggered for low levels of excitation. This may form the basis of pathological burst generation and transmission in parkinsonian conditions.

## Introduction

The basal ganglia are thought to primarily exert their motor function in mammals by projecting to cerebral cortex via motor thalamus, specifically the VA/VL thalamic nuclei in primates, and VM and rostroventral VA/VL in rodents (Sakai et al., 1998; Kuramoto et al., 2009, 2011). The classic functional model of this basal ganglia loop back to cortex is that GABAergic basal ganglia output from the internal segment of pallidum (GPi) and substantia nigra (SNr) reduces the spike rate of motor thalamus roughly in proportion to the spike rate of basal ganglia output (Alexander et al., 1986; Delong, 1990). Due to the high baseline rate of spiking in GPi/SNr *in vivo* (Delong, 1971), this results in a tonic inhibition of movement related activity in motor thalamus. A pause in the tonic basal ganglia output would lead to disinhibition of motor thalamus that results in the activation of movement or “action selection” (Gurney et al., 2001; Humphries et al., 2006). This model of “classic” thalamic inhibition has recently been challenged by results showing that strong inhibitory inputs from the basal ganglia could result in rebound activation of thalamic neurons. This possibility has been primarily supported by data from the basal ganglia output to the DLM thalamus in the songbird vocal learning circuit showing rebound activation after individual inhibitory inputs in brain slices (Person and Perkel, 2005) or anesthetized birds (Person and Perkel, 2007). If generally true, this mode of synaptic integration would invert the sign of basal ganglia output, and result in specialized burst activation of thalamus following basal ganglia input. The songbird system is somewhat specialized, however, in that a single strong basket-type synapse connects a basal ganglia output cell to a DLM thalamic neuron (Luo and Perkel, 2002), which leads to precisely timed strong inhibition (Luo and Perkel, 1999a). Nevertheless, the rodent and primate connection from the SNr to motor thalamus also consists of strong perisomatic clusters of synapses, that likely form a “driver” type of input (Bodor et al., 2008). In addition, thalamic neurons in mammals as well as songbirds show a strong T-type calcium current, which produces post-hyperpolarization rebound bursts (Jahnsen and Llinas, 1984a,b). Such T-type bursting is ubiquitous in thalamic recordings both in sleep and in active states (Ramcharan et al., 2000; Llinás and Steriade, 2006). In this study we use an optogenetic approach to selectively activate basal ganglia input to motor thalamus in order to determine whether in mammals basal ganglia output is likely to operate via a rebound coding mechanism, or whether the classic model of spike inhibition better describes the effect of the basal ganglia on thalamus. This question is fundamental to our understanding of basal ganglia effects on cortical activity, and has been flagged as one of the most significant unknowns regarding basal ganglia function in prominent recent reviews of the field (Graybiel, 2005; Nambu, 2008).

## Materials and methods

All animal procedures followed approved Emory University IACUC protocols. Adult male C57BL/6 mice (Jackson Labs) of 3–7 months of age were stereotactically injected with AAV5-Syn-ChR2(H134R)-EYFP or AAV1-Syn-ChR2(H134R)-EYFP (200–500 nl with NanojectII; Drummond Scientific) into the SNr pars reticulata under isoflurane anesthesia. At 3–5 weeks following injection mice were sedated in isoflurane and anesthetized with ketamine/xylazine (200 mg/kg ketamine and 40 mg/kg xylazine i.p.). After complete cessation of reflexes mice were perfused transcardially with cold extracellular saline modified to protect from excitotoxic processes following hypoxia. The media contained (in mM): (Sucrose 235.4 (or Choline Chloride 117.7); KCl 2.5; NaH_2_PO_4_ 1.25; MgCl 7; NaHCO_3_ 26; D-Glucose 10; L-ascorbate 1; NaPyruvate 3). The brain was removed quickly and slices cut in ice-cold solution at 250 μm with a vibratome (Microm HM 650). Slices were incubated in extracellular medium (choline or sucrose replaced with NaCl 124) for 60–90 min at 34°C and afterward gradually returned to room temperature. Despite these precautions viable slices were only obtained from about 50% of perfused mice, as adult mouse thalamic tissue poses a great challenge due to extensive myelination.

Slices were transferred to a visualized whole cell recording setup, and fluorescent areas in motor thalamus due to EYFP expression were sought out for visualized whole cell patch recordings. Recordings from visualized thalamus neuron somata were obtained under a 60× Olympus water immersion lens using pipettes pulled from 1.5 mm OD borosilicate glass on a Sutter P-97 puller (Sutter Instruments, Novato, CA). Pipettes were filled with solution containing (in mM): 140 K-gluconate, 6 NaCl, 2 MgCl2, 4 Na2ATP, 0.4 Na3GTP, 0.2 EGTA, 5 glutathione, 0.5 spermine, 0.02 Alexa-568, and 10 HEPES, pH 7.3 with KOH. The temperature of the slice chamber was maintained at 32°C with a feedback controller and a Peltier element (Luigs and Neumann, Ratingen, Germany). Optical stimulation of ChR2 was performed either with a blue laser light source (Shanghai Dream Lasers, SDL-473-060T) through a 200 μm glass fiber pointed at the motor thalamus at a distance of 1–3 mm or with whole field light flashes at 60× magnification with the fluorescent imaging light source of our microscope (120 W mercury bulb, X-Cite) filtered to pass blue light. In the second case a Uniblitz shutter was used to control exposure times. In some cases both light sources were used on the same recorded neurons and the elicited IPSPs were found to be equivalent. However, sub-millisecond exposure times were only possible using the laser light source. We used single light pulses as well as paired pulses and light pulse trains of up to 20 Hz frequency for stimulation in order to explore post-inhibitory effects after periods of inhibition of varying duration. While faster than 20 Hz stimulation might more naturally mimic SNr firing frequencies *in vivo*, due to desensitization and the relatively slow deactivation time constant of ChR2 (>10 ms at physiological pH) (Nagel et al., 2003) such faster stimulation frequencies do not reliably trigger spikes (Boyden et al., 2005). These properties of ChR2 also prevented us from characterizing the properties of short term plasticity in the nigral input to thalamus, as frequencies sufficiently high to test for facilitation or depression while maintaining reliable stimulation strength for each input could not be achieved. Electrophysiological data were obtained using an Axon Multiclamp 700 B amplifier (Molecular Devices) and a customized Labview (National Instruments) software interface that allowed flexible current injection and optical stimulation schedules (see Results). Cell attached recordings were obtained in voltage clamp, and a shift away from zero holding current was taken as partial break-in. A fluorescent dye (AlexaFluor-568) was included in the patch pipette so that we could visually confirm that the cell cytoplasm had not been infused with dye during cell-attached patch recordings. For a subset of recordings glutamate puffs were delivered via a second patch electrode connected to a Picospritzer (usually ≤ 5 psi pressure, 3–5 s). Slices were saved and fixed in 10% buffered formaldehyde (Sigma) for later visualization and digital imaging. In some cases pictures of recorded cell bodies were also obtained during recording using a Dage MTI camera attached to the recording setup. Data were analyzed using Matlab (Mathworks, Inc.).

## Results

**Figure 1:**
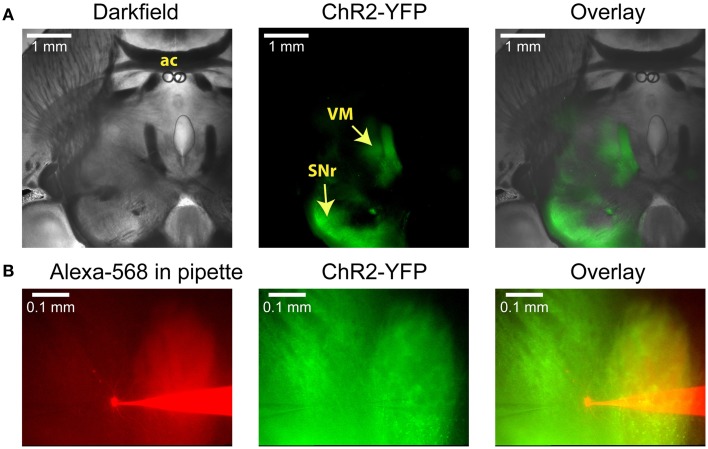
**Anatomical images illustrating a typical experiment. (A)** Low-power images of a horizontal slice containing both the SNr and the VM. This level corresponds to Plate 144 in the Paxinos atlas (Franklin and Paxinos, 2008). AAV5 virus carrying YFP-tagged ChR2 under the control of the synapsin promoter was injected into the SNr, where it infected neurons and resulted in YFP fluorescence both in the surrounding tissue and in the VM thalamus. **(B)** Higher-power view shows the recording pipette and a VM neuron filled with the red dye Alexa-568 (left), the YFP fluorescence that marks ChR2 expression (middle), and the overlay (right). A glutamate puffer pipette can be seen on the left side of the middle image.

In order to selectively stimulate nigral inputs to motor thalamus during whole cell recordings of VM neurons we injected an AAV vector carrying YFP-tagged channelrhodopsin-2 (ChR2) into the SNr of adult mice 3–5 weeks prior to preparing brain slices (see Materials and Methods). In horizontal brain slices YFP was clearly visible in the SNr on the side of the injection, in connecting fiber bundles, and in motor thalamus (Figure 1A). Whole cell recordings were obtained from the thalamic area with dense terminal YFP labeling (Figure 1B). This area consisted primarily of VM and adjacent VL as defined in the Paxinos mouse atlas (Franklin and Paxinos, 2008) in agreement with the “inhibitory afferent dominant” (IZ) zone found in rats (Kuramoto et al., 2009, 2011; Nakamura et al., 2014).

### Nigral IPSP post-synaptic potentials and currents in motor thalamus

**Figure 2:**
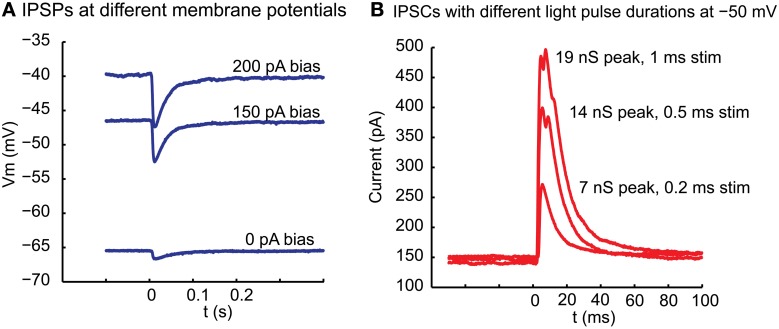
**Inhibitory potentials and underlying currents resulting in motor thalamus from local ChR2 activation in nigral termination areas. (A)** At the time indicated by 0 a 10 ms light pulse was delivered from a blue laser light source to the slice via a glass fiber. The fiber was held with a micromanipulator and the tip manually placed close to the brains slice with the light cone pointed at VM. The baseline membrane potential at the time of stimulation was controlled with a bias current injection through the recording electrode. **(B)** Outward currents elicited with a light pulse of varying duration between 1 and 0.2 ms in voltage clamp at −50 mV holding potential. Note that the response is graded with stimulus duration and shows multiple peaks at longer stimulus duration. Even with the shortest stimulus duration, however, failures were not observed, arguing against this ~100 pA response being carried by a single synapse. The response latency from light stimulus to IPSC onset was 3.2 ms in this neuron.

Stimulation of ChR2 with blue light (see Materials and Methods) at the location of whole cell recordings in thalamus reliably triggered an inhibitory post-synaptic potential (IPSP) in current clamp (Figure 2A). This IPSP had a reversal potential near −70 mV, which was in agreement with the chloride Nernst potential of −68 mV calculated from the concentration used in our intracellular and extracellular solutions at 32°C. Similarly, for a sample of 5 neurons for which the same light stimulus was repeated at 2 different potentials by applying a bias current in current clamp, the reversal potential calculated from a linear fit was −69 + −2.6 mV. Of particular interest is the amplitude distribution of basal ganglia inhibitory inputs to thalamus, as rebound responses would be most likely observed following large amplitude inhibitory events. The same sample of 5 neurons recorded at multiple membrane potentials showed a mean interpolated amplitude of IPSPs of −8.0 mV at a potential of −50 mV at maximal stimulation strength. The amplitude of inhibitory post-synaptic currents (IPSC) in voltage clamp was graded with stimulation strength and responses to larger stimuli could show multiple peaks (Figure 2B), possibly due to triggering action potential bursts in pre-synaptic fibers due to the slow deactivation of ChR2 (Nagel et al., 2003). Previous studies have indicated that doublet action potential firing is a common consequence of brief ChR2 activations (Gunaydin et al., 2010). In 6 recordings tested in voltage clamp, the peak conductances obtained with our stimuli ranged from 2.7 to 19.4 nS (mean 8.6 nS). In three cases where multiple stimulation intensities were employed in voltage clamp for 1 ms stimuli by adding neutral density filters of different opacity to the light path from the UV lamp we found ratios of 2.7 (93 vs. 249 pA), 5.5 (20 vs. 110 pA), and 13.2 (7.5 vs. 99 pA) between the largest and smallest average IPSC amplitude that was elicited for the smallest and largest stimulation intensity used. Note, however, that limitations of this technique do not allow us to ascertain the size of a single nigral input, nor can we be certain that all nigral axons express sufficient ChR2 to initiate an action potential for our maximal stimulation strength. Nevertheless, these results are consistent with the recruitment of a small number of unitary inhibitory connections with multiple active zones as would be expected from anatomical findings (Bodor et al., 2008). The exponential decay time constant of single peaked IPSCs elicited with small stimuli ranged between 12 and 16 ms (mode: 14 ms, 4 neurons).

### Low-threshold spike bursts in motor thalamus

**Figure 3:**
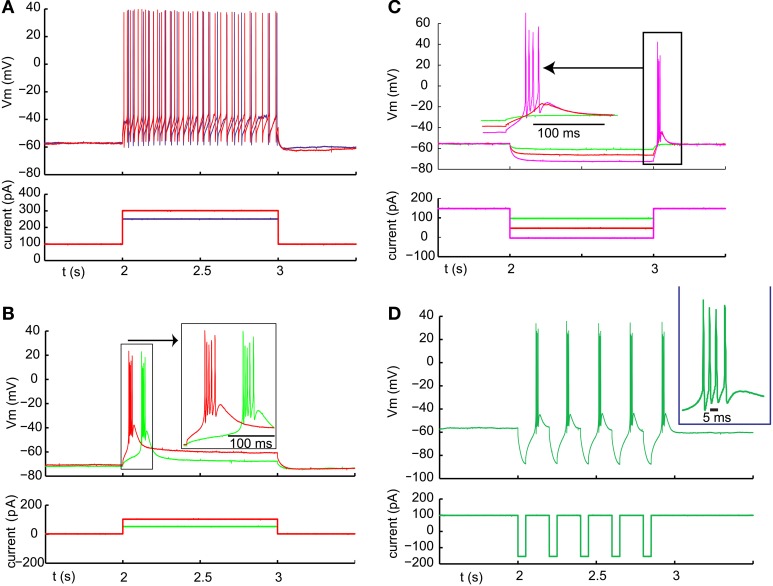
**Typical examples of LTS burst properties from IZ thalamic neurons. (A)** Inward current injection from a depolarized baseline results in continuous single spike mode with little adaptation. The spike rate increases steeply with injected current amplitude (250 pA resulting in 22 spikes /s (blue trace) and 300 pA in 32 spikes/s (red trace). **(B)** Inward current injection from the resting potential results in an LTS burst with a shorter latency for larger current injection (red trace). **(C)** Same cell as **(B)**. An LTS burst could also be triggered as a post-hyperpolarization rebound without an additional depolarizing current injection when the hyperpolarization was strong enough (see Results). **(D)** Same cell as **(A)**. A train of LTS bursts elicited by a train of hyperpolarizing pulses.

In order to fire rebound spike bursts with basal ganglia input, neurons in the IZ zone of thalamus would need to have a sufficient amount of T-type calcium current with inactivation properties that allow de-inactivation during nigral IPSPs or IPSP bursts. While thalamic neurons in general are known to have a substantial T-type calcium current (Llinas and Jahnsen, 1982), regional thalamic differences in rebound burst firing properties and T-type channel density have been observed (Wei et al., 2011). Rebound properties of IZ thalamus neurons in mice have not been previously determined to our knowledge. All of our IZ recordings were obtained from adult mice to exclude a developmental immature stage. Successful whole cell IZ recordings (*N* = 13) were confirmed by eliciting IPSPs with optical nigral terminal stimulation (see Methods). IZ neurons all showed a hyperpolarized non-spiking baseline, and typically required a current injection of 100–300 pA to elicit a tonic single-spike mode (Figure 3A). Note that the baseline resting membrane potential was generally hyperpolarized enough that a small depolarizing current injection would trigger a single low-threshold spike (LTS) burst (Figure 3B; observed in 7 of 7 IZ neurons analyzed with this stimulus, average Vm was −67 mV). Therefore, the presence of an LTS burst did not necessarily require a preceding inhibitory input as the resting potential allowed sufficient de-inactivation of T-type channels to trigger such a burst with excitation. However, in the awake animal a less hyperpolarized membrane potential may be expected, and therefore we tested how much hyperpolarization was needed to elicit a rebound LTS burst when starting from a depolarized baseline of around −55 mV (Figure 3C). We found that hyperpolarization for 1 s from −55 to −60 mV was insufficient to trigger a rebound (Figure 3C, red trace), whereas a stimulus resulting in −65 to −70 mV did result in a rebound in the same neurons (Figure 3C, magenta trace, *N* = 5 neurons tested). Repetitive shorter negative current injection pulses of 50 ms resulted in a train of LTS bursts (Figure 3D). This suggests that strong, repetitive inhibitory basal ganglia inputs to IZ neurons may be able to drive LTS bursts.

### Direct testing of whether optically activated inhibitory inputs could elicit LTS bursts

**Figure 4:**
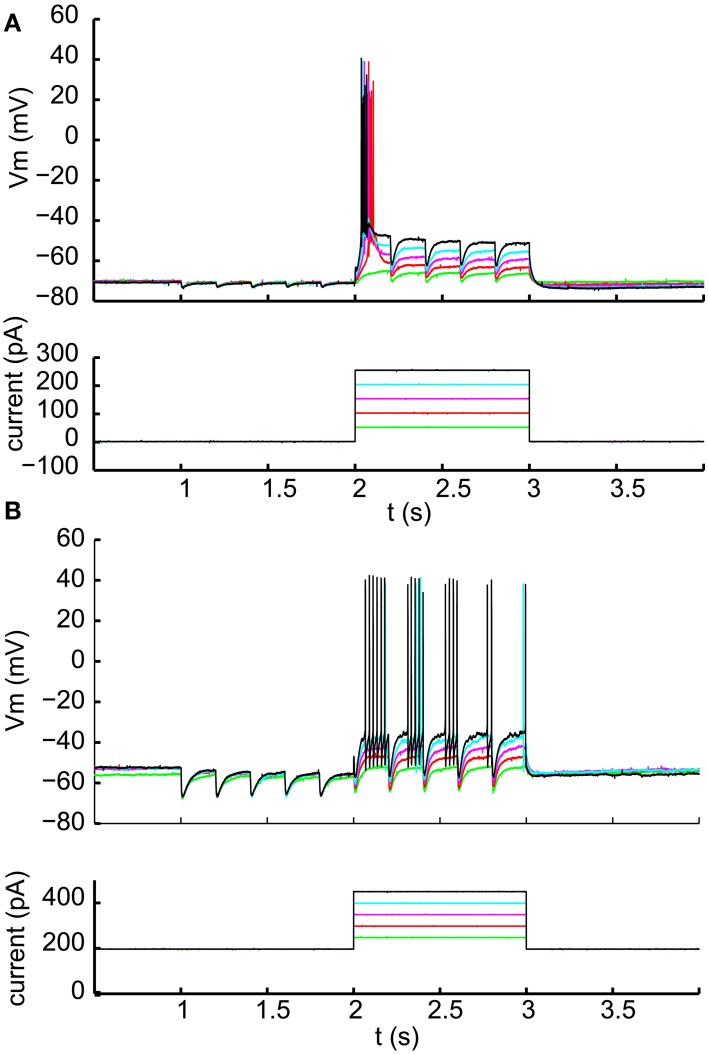
**Failure of nigral single IPSPs triggered by 5 ms light pulses to elicit LTS bursts at any membrane potential. (A)** Trains of IPSPs at 5 Hz at rest and during varying levels of depolarizing current injection as shown below the voltage traces. **(B)** Trains of IPSPs at 5 Hz during a depolarized baseline and subsequent additional inward current injection.

We optically activated nigral input in single pulses or trains while recording from IZ neurons. We manipulated the level of IZ neuron baseline depolarization with current injection in order to investigate whether rebounds could be triggered following synaptic hyperpolarization. The result was that inhibitory nigral input triggered with single 1–10 ms light pulses never elicited a rebound (Figure 4, *n* = 14). At hyperpolarized membrane potentials the driving force of inhibition was too small to allow substantial further hyperpolarization (Figure 4A, left side). While a depolarizing input such as the onset of current injection could trigger an LTS burst (Figure 4A, middle), individual IPSPs at a more depolarized potential were again too small and too brief to allow sufficient T-type channel de-inactivation to result in post-inhibitory rebound spikes (Figure 4A, right). When the cell was depolarized sufficiently to be in the single spiking mode, individual nigral inputs led to pauses in spiking (Figure 4B, seen in 7 of 8 cells tested, 1 cell showed an IPSPs too small to lead to a discernible spike pause).

**Figure 5:**
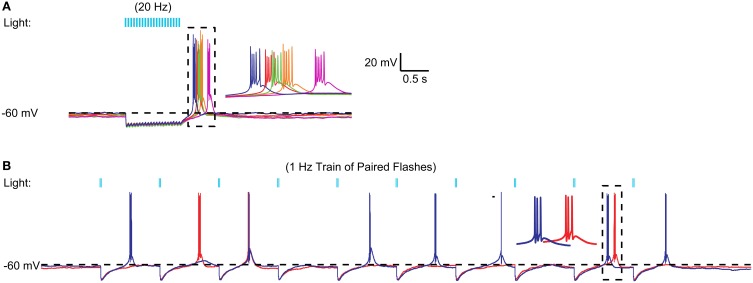
**Example of high frequency optical stimulation trains and brief stimulation bursts to elicit LTS bursts. (A)** A 1 s 20 Hz train of IPSPs triggered by 5 ms light pulses resulted in an LTS rebound burst at varying latencies. A 20-Hz train of 50 IPSPs triggered by 5 ms light pulses consistently resulted in LTS bursts in a VM neuron. Five different trials are overlaid to illustrate the trial-to-trial variability in the LTS latency. **(B)** Same neuron. Paired pulses (2 ms pulses, 20 ms interval) elicited LTS rebounds for some paired stimuli. These LTS rebounds consisted of 1–3 spikes.

We then tested whether stronger activation of nigral inputs could result in sufficiently deep and long hyperpolarization to elicit rebound activity. To this end we used short sequences of 20 Hz light stimulation or paired pulses of stimulation with an interstimulus interval of 20 ms. Indeed, in some recordings tested with fast input trains (Figure 5A) or input doublets (Figure 5B) we noted that stereotypical LTS rebound bursts could follow such input sequences. This was observed in 3 of 10 recordings tested with 20 Hz stimulus trains or 50 Hz paired pulses. Because this mode of optogenetic activation resembles bursty nigral input patterns, which are a prominent feature in Parkinson's disease, these results so far suggest that a normal regular spiking activity mode of nigral GABAergic neurons does not result in rebound burst transmission in the motor thalamus, while such a mode may be partially occurring in the parkinsonian condition. In agreement with this finding thalamic activity in waking Parkinson's patients has shown a high degree of LTS bursting (Hodaie et al., 2006) and a role for thalamic rebound bursting in generating tremor in PD patients has been hypothesized (Pare et al., 1990).

### Interaction of optogenetic nigral input inhibition and glutamatergic excitation in IZ neurons

**Figure 6:**
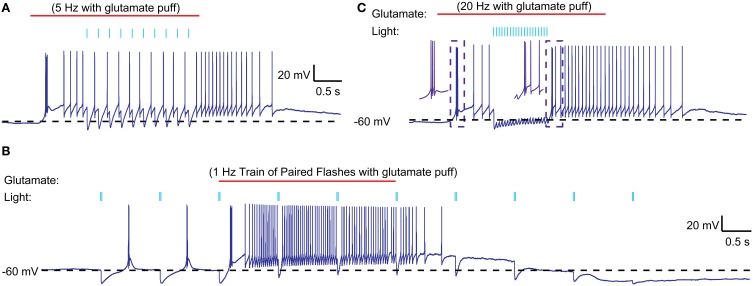
**Interaction of optically stimulated nigral IPSPs with glutamatergic excitation. (A)** Glutamate (0.5 mM) was “puffed” at the recorded neuron for 3 s (red line) with a patch pipette brought in close proximity. During the single spike mode triggered by glutamate excitation a 5 Hz train of 10 ms light pulses (indicated by blue bars) causes pauses in spiking but not LTS rebound burst is evoked. **(B)** Paired light pulses (2 ms pulse width, 20 ms interval) before glutamate excitation can cause LTS rebound bursts (as shown in Figure 5), but the same paired pulses during glutamate excitation result in spike pauses only. **(C)** A 20 Hz train of 5 ms light pulses results in a prolonged pause during glutamate excitation and a weak LTS rebound burst (2 spikes) afterwards.

Because our depolarizing current injections were at the soma, they cannot reflect possible dendritic interactions between excitatory post-synaptic potentials (EPSPs), IPSPs, and dendritic T-type channels in the control of rebound LTS activity. To directly test for such interactions, we applied low-pressure, sustained glutamate puffs (3 or 4 s, 0.5 mM glutamate) to the dendritic area of IZ neurons during whole cell recording to trigger receptor-evoked spiking. This method very reliably evoked spike trains of several seconds duration (Figure 6). The spike trains frequently started with an LTS burst, again indicating that the resting potential of IZ neurons in brain slices is sufficiently hyperpolarized to allow sufficient de-inactivation of T-type current such that excitation can trigger LTS bursts. However, during the ensuing tonic glutamate-driven single spike period, optogenetically driven nigral input led to regular inhibition for single inputs (Figure 6A) and paired pulse inputs (Figure 6B). This was seen in all 5 neurons tested with this input paradigm. Only when fast trains of strong nigral input were stimulated did the synaptic inhibition lead to sufficient hyperpolarization to occasionally allow an LTS rebound (Figure 6C). This was observed in 2 of 5 cells tested, while the other 3 only showed single spike suppression. It should be noted that the optogenetic light stimulation used here illuminated a large area around the recorded IZ neuron and is therefore likely to simultaneously activate a substantial proportion of all nigral inputs to the cell, a condition not expected to occur in the desynchronized normal state of nigral input *in vivo*, but possibly more common in the parkinsonian condition where nigral activity has been observed to become more bursty (Murer et al., 1997; Wichmann et al., 1999) and more synchronized between neurons (Avila et al., 2010; Brazhnik et al., 2012). Hence, in mice, LTS rebounds are unlikely to occur in the single-spike mode of thalamic activity as a consequence of desynchronized regular basal ganglia inputs, but may in some cases be triggered by synchronized bursts as observed in parkinsonian conditions.

**Figure 7:**
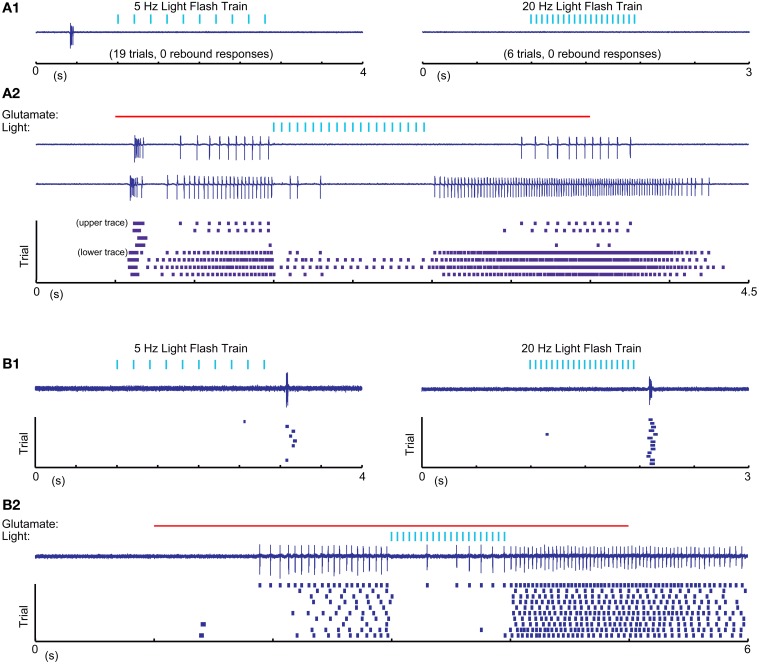
**Cell attached recordings with glutamatergic *excitation* and optical nigral input stimulation. (A1,B1)** 5 Hz 10 ms and 20 Hz 5 ms light pulse trains result in LTS rebounds in one cell **(B)**, but not another **(A)**. Purple traces show recordings of extracellular spike currents and raster histograms below show spike times for repeated application of the same stimulus combination. **(A2,B2)** The same optical stimuli delivered during a 4 s 0.5 mM glutamate application result in pronounced spike pauses of glutamate-stimulated activity without a terminating LTS rebound burst in either cell. The strong initial rebound of cell **(A)** but not cell **(B)** with glutamate application suggests that this cell had a more hyperpolarized resting membrane potential than cell **(B)**. A more depolarized baseline of cell **(B)** is also suggested by it showing LTS rebounds following IPSP trains as our intracellular recordings only resulted in such rebounds when the cell was considerably depolarized above ECl.

One possible explanation for the relative inability of SNr inputs to produce LTS rebounds in this study is that the chloride reversal potential with our whole-cell recordings solutions may be more depolarized compared to the native state of IZ neurons. This would reduce the depth of IPSPs and diminish the amount of T-type calcium channel de-inactivation produced by SNr stimulation. To address this concern we performed cell-attached patch recordings, enabling us to monitor the spiking activity of IZ neurons with natively-determined chloride reversal potentials. IZ neurons in cell-attached mode were generally non-spiking except rare occurrences of spontaneous LTS bursts (Figure 7A1), consistent with the expected hyperpolarized resting membrane potentials. As in our whole cell recordings, optogenetic input stimulation rarely led to LTS bursts (Figure 7A1), even if in the same neuron the same stimulation parameters were highly effective at inhibiting glutamate puff—evoked spike trains (Figure 7A2) and LTS bursts were present at the onset of such glutamate puff—evoked activity (*N* = 5 of 5 neurons tested). As also observed in whole cell recordings, in a subpopulation of neurons (2 of 5 neurons tested) trains of optogenetic light stimulation could evoke an LTS burst from rest (Figure 7B1). Nevertheless, during glutamate evoked spiking these cells also showed traditional spike inhibition without LTS rebounds. These recordings fully confirm the findings obtained with whole cell methods.

**Figure 8:**
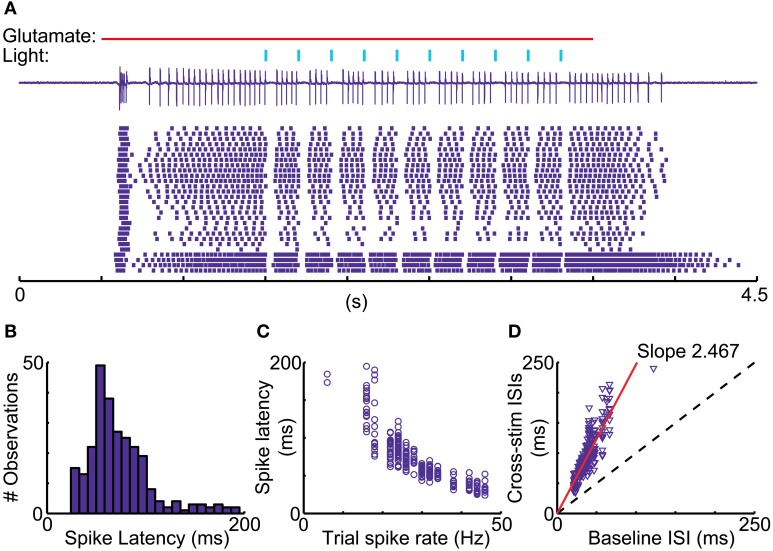
**Variable duration of spike pauses with nigral IPSP. (A)** Sample cell-attached current trace and raster plot of 28 stimulus repetitions for a 5 Hz train of 10 ms light pulses during glutamate excitation. Note the lack of alignment of the first spike following pauses. **(B)** Latency histogram of first spike after the onset of optical light pulses. **(C)** Scatter plot of first-spike latency against the baseline spike rate during each trial measured in the 500 ms before the first light pulse. **(D)** Scatter plot of ISI, in which the stimulus occurs, vs. mean trial baseline ISI.

A recent report in songbird motor thalamus indicates that basal ganglia input *in vivo* may lead to entrainment of spiking with pallidal inputs in the presence of a constant excitatory drive (Goldberg et al., 2012). We looked for such entrainment during glutamate puff-evoked activity, which should be apparent by an alignment of spikes at a constant delay following optical stimulation of inhibitory input. However, repeated stimulation trains revealed highly variable timing of the first spike after optically triggered inhibition (Figure 8). The observed variability in first-spike latency from the time of stimulation (Figure 8B) was partially accounted for by the baseline spike rate prior to stimulation, but even for matching spike rates a considerable variability in latency remained (Figures 8C,D). This result was obtained in all IZ neurons tested (*N* = 5).

## Discussion

### Experimental limitations

Several limitations of the present *in vitro* study need to be considered when interpreting the data with respect to *in vivo* function of thalamic coding of basal ganglia input. First, due to slicing procedures, slice recovery, lack of neuromodulation *in vitro*, partial deafferentation, altered chemical milieu, etc, synaptic properties may be different from normal thalamic processing in the awake mouse. These are limitations inherent in all slice work, and extrapolation to the awake condition should be best understood as a hypothesis to be examined in the awake condition when possible. Second, the recordings obtained in our study were not of sufficient numbers, and the anatomical specification of recording sites was not accurate enough, to assess the potential for heterogeneity between different sub-areas of the IZ thalamic zone that can be expected on anatomical grounds (Sakai et al., 1998). In addition, plasticity processes could allow a small number of thalamic neurons to receive specific functional populations of strong basal ganglia synapses, which we might have missed.

While the benefit of an optogenetic approach in specifically eliciting inhibition from nigral inputs as opposed to electrically stimulating a mix of nigral and nRT inputs is obvious, optogenetics comes with its own sets of limitations. Specifically with ChR2 expression effected through stereotactic AAV viral vector injections, a certain amount of variability in the completeness and uniformity of expression can be expected. Therefore, small synaptic response sizes in any given slice preparation could well be due to partial ChR2 expression in the afferents of recorded neurons, and such responses do not signify an equally small total synaptic conductance in the nigral inputs that was previously present *in vivo*. On the other hand, synchronous stimulation of all afferents is also not a physiological stimulus, and the maximal response sizes observed may well be larger than any synchronous inputs usually present *in vivo*. Therefore, rebound responses observed in slices may not carry over to the *in vivo* situation. Optogenetics also has limitations when it comes to traditional methods such as analyzing miniature PSCs or minimal stimulation, as minis cannot be optically evoked, and minimal stimulation cannot be expected to reliably activate the same fiber repeatedly due to the relatively large spatial extent of the optical input. These limitations present an obstacle to fully characterizing the properties of single connections.

Nevertheless, we believe that despite these limitations our data present an important first glimpse at the synaptic properties and functional role of nigral GABAergic input to the IZ zone of motor thalamus as discussed in the following paragraphs.

### Properties of synaptic connections from the SNr to the IZ zone in motor thalamus

In this study we took advantage of optogenetic methods to selectively stimulate the basal ganglia input to motor thalamus in a mouse slice preparation. This allowed a characterization of the synaptic properties of this important pathway for the first time. We focused our efforts on adult mice (4–8 months age) in order to avoid describing a transient developmental state of this pathway, as GABA receptor subtypes undergo developmental regulation (Fritschy et al., 1994) and inhibitory synaptic conductance properties in mouse thalamus have been shown to mature at least through day p27 (Peden et al., 2008). GABA_A_ conductance properties have been previously determined for the ventral basal nucleus (VB) of thalamus in rat (Cox et al., 1997; Zhang et al., 1997) and mice (Peden et al., 2008; Zhang et al., 2010; Laudes et al., 2012; Herd et al., 2013), where GABA synapses in rodents are almost exclusively associated with inputs from the reticular thalamic nucleus (nRT). Miniature IPSCs in 14–19 day old mice were recorded from VB with a mean amplitude of 24 pA and a decay time of 9.2 ms at 23–25°C (Laudes et al., 2012) and 22.3 pA and 4.4 ms decay at 32–34°C. (Zhang et al., 2010), suggesting a strong dependence of decay kinetics but not maximal IPSC amplitudes on temperature. Voltage clamping was done with a driving force of 65–70 mV in these studies, indicating a quantal peak conductance of 0.3–0.4 nS, similar to the smallest conductance values found in our optogenetic stimulation responses with small stimuli. Electrical stimulation of nRT fibers results in evoked IPSCs with a mean amplitude of 394 pA (5.6 nS) for minimal stimulation and 1093 pA (15.6 nS) for maximal responses (Zhang et al., 2010). Paired recordings of mouse nRT and VB neurons showed a unitary connection strength with an average of 142.6 pA, corresponding to 4.1 nS in this study due to recordings with physiological Cl^−^ in the recording pipette and voltage clamping with a 35 mV driving force (Herd et al., 2013). In the present study we observed optically evoked IPSCs of 2.7–19.4 nS, with the larger IPSCs involving the stimulation of multiple nigral fibers whereas the smaller single peaked IPSCs likely correspond to single fiber stimulation. Even smaller presumed single fiber stimulation responses were seen for low intensity light stimulation in 2 cases. Therefore both the presumed single fiber connection of ~1–3 nS and the maximal connection of about ~19 nS present similar values to the nRT to VB connection. Given these values and our ratios of observed minimal and maximal response amplitudes for different stimulation intensities showing values between 3 and 13, our data suggest that a single VM neuron may be contacted by between 3 and 13 SNr fibers. Note that especially the low end of this range may be an underestimate because in any given experiment we may not have achieved sufficient ChR2 expression in all nigral axons to trigger an action potential with light stimulation in the terminal area.

The average decay time of the IPSCs observed in IZ neurons in the present study was 14 ms, compared to 9 ms in the VB thalamus of mouse pups and 3.4 ms in the VB thalamus of adult mice (Peden et al., 2008). The slower decay time in our recordings is unlikely due to dendritic filtering as nigral synapses are frequently located close to the soma (Bodor et al., 2008). An increase in decay time from 3.4 ms to 14 ms denotes a 4-fold increase in charge transfer for a single IPSC, thus substantially contributing to a high strength of nigral synaptic transmission. While our study did not attempt to determine the GABA receptor subunits situated at nigral synapses in thalamus, previous studies indicate that different thalamic nuclei can show substantially different GABA_A_ IPSC decay kinetics based on a different subunit composition (Huntsman and Huguenard, 2000; Browne et al., 2001).

### Functional implications

A key question our study addressed was whether the transmission of nigral output to cerebral cortex via IZ thalamus involves post-inhibitory rebound LTS bursts as has been observed with basal ganglia inhibition of songbird motor thalamus (Person and Perkel, 2005, 2007; Leblois et al., 2009) or predominantly suppresses thalamic spike activity as is posited by the classical rate model of basal ganglia function (Albin et al., 1989; Delong, 1990; Wichmann and Delong, 1996). We found no support for the rebound hypothesis for the expected regime of normal basal ganglia function, in which basal ganglia output spiking is continuous and desynchronized (Raz et al., 2000) and motor thalamus is in an irregular single spiking mode with few neurons also showing bursts (Anderson and Turner, 1991; Vitek et al., 1994; Macia et al., 2002). Essentially, we found that in the slice preparation, nigral inhibition of spiking thalamic neurons was too small and too brief to allow T-type calcium current to de-inactivate for a subsequent rebound LTS burst (Figures 4, 6, 7). While there is evidence for multiple synaptic inputs from individual nigral axon terminals to single post-synaptic neurons in rodent IZ thalamus (Bodor et al., 2008), this configuration did not produce giant unitary IPSCs in our data, likely because not all active zones in these terminals release transmitter for each stimulus. In fact, the size of ~1–3 nS for small presumed single fiber optically evoked IPSCs is consistent with the release of about 3–10 quanta of 0.3 nS amplitude. The case in songbird is different in that a thalamic IZ neuron is contacted only by a single basal ganglia input axon, which forms a strong basket of synapses around the soma (Luo and Perkel, 1999a,b), thus allowing a stronger potential for LTS rebound bursts. Even with this unusual anatomical arrangement, recent evidence indicates that in awake songbirds, thalamic neurons are sufficiently depolarized that most basal ganglia inputs do not produce post-inhibitory rebounds (Goldberg et al., 2012).

In our preparation it was also unusual to elicit a post-inhibitory rebound from the hyperpolarized resting potential (Figure 4), because the resting potential was very close to the reversal potential of chloride, thus leaving almost no driving force for further hyperpolarization. However, in a proportion of both whole cell and cell attached recordings (Figures 5–7) neurons did respond with a post-inhibitory rebound after trains or paired pulses of optical nigral input stimulation. Therefore, in conditions in which IZ thalamic neurons receive bursty and synchronized input and are weakly depolarized, a regime for post-inhibitory rebounds opens up. The conditions of bursty and synchronized basal ganglia output activity have been characterized as a hallmark property of parkinsonian pathophysiology (Wichmann and Dostrovsky, 2011), and in fact a high degree of LTS bursting is observed in human PD patients (Magnin et al., 2000). Therefore, excessive rebound LTS bursting of IZ thalamic neurons in the awake condition might contribute to motor dysfunction in parkinsonism, notably the generation of tremor (Pare et al., 1990). Not every LTS burst is necessarily triggered by basal ganglia input, however, as excitatory inputs at the resting potential of IZ thalamic neurons can also trigger LTS bursts (Figures 6, 7). Motor cortical activity in MPTP lesioned parkinsonian primates becomes also more bursty than that of controls (Goldberg et al., 2002), and the motor cortex has substantial connections with IZ thalamic neurons (Kultas-Ilinsky et al., 2003). Therefore, both excitatory and inhibitory input patterns in the parkinsonian condition could contribute to increased LTS burst firing in this condition. Future studies using whole cell recordings from thalamus in awake, 6-OHDA lesioned rodents could directly determine the relative contribution of inhibition and excitation and their possible phase locking in the control of LTS bursting in IZ thalamus. Phase locked motor cortical and nigral activity in the beta frequency band has recently been reported during walking related activity in 6-OHDA lesioned rats (Brazhnik et al., 2012).

We did find that optical nigral stimulation reliably induced a short pause in IZ thalamic firing, and that light pulse trains simulating a period of increased nigral activity *in vivo* could shut down glutamate elicited spiking completely (Figures 6, 7). In contrast to a previous study in songbird (Goldberg et al., 2012), however, we did not observe that spikes following nigral inhibition were entrained to the timing of inhibitory input. A possible explanation for this difference is that inhibitory input in the mammal through multiple synapses is not as temporally precise as the single 1:1 pallido-thalamic synapse in songbird. Therefore our findings are most in agreement with the traditional rate coding model of normal basal ganglia function, in which nigral firing rates are translated to an opposite firing rate control of IZ thalamic neurons. SNr neurons show sustained firing rates with an average of 28 Hz in rats (Ruskin et al., 1999) and 66 Hz in monkeys (Wichmann and Kliem, 2004). On this background, distinct rate increases and decreases are found to be closely locked to sensory and motor aspects of behavior (Wichmann and Kliem, 2004). Similarly, activity in the internal pallidum (GPi), the other basal ganglia output nucleus projecting to motor thalamus, shows a high baseline rate of desynchronized firing with strong behaviorally locked rate changes that can be proportional to motor parameters (Georgopoulos et al., 1983). Motor thalamus similarly shows motor-related rate changes (Anderson and Turner, 1991; Vitek et al., 1994). Based on our findings, we expect that these rate changes in basal ganglia output nuclei will cause inverted rate changes in motor thalamus in agreement with the classical model of basal ganglia function. Interestingly, a similar rate coding model has recently found strong support for the output from the cerebellum (Walter and Khodakhah, 2006, 2009).

Thalamic activity in normal animals is not entirely devoid of LTS bursts (Sherman, 2001), however, and for sensory areas of thalamus such bursts have been associated with specific alerting events that may occur when the thalamus is temporarily in a hyperpolarized non-alert state (Sherman and Guillery, 2002; Stoelzel et al., 2009; Bereshpolova et al., 2011). A similar type of specialized event may also explain the presence of rare LTS spikes in normal waking conditions in motor thalamus.

## Author contributions

Jeremy R. Edgerton obtained slice recordings in cell attached and whole cell mode, analyzed data, and critically edited the manuscript. Dieter Jaeger obtained slice recordings in whole cell mode, analyzed data, and wrote the manuscript.

## Conflict of interest statement

The authors declare that the research was conducted in the absence of any commercial or financial relationships that could be construed as a potential conflict of interest.

## References

[B1] Albin R. L. Young A. B. Penney J. B. (1989). The functional-anatomy of basal ganglia disorders. Trends Neurosci. 12, 366–375 10.1016/0166-2236(89)90074-X 2479133

[B2] Alexander G. E. Delong M. R. Strick P. L. (1986). Parallel organization of functionally segregated circuits linking basal ganglia and cortex. Ann. Rev. Neurosci. 9, 357–381 10.1146/annurev.ne.09.030186.002041 3085570

[B3] Anderson M. E. Turner R. S. (1991). Activity of neurons in cerebellar-receiving and pallidal-receiving areas of the thalamus of the behaving monkey. J. Neurophysiol. 66, 879–893 1753292 10.1152/jn.1991.66.3.879

[B4] Avila I. Parr-Brownlie L. C. Brazhnik E. Castaneda E. Bergstrom D. A. Walters J. R. (2010). Beta frequency synchronization in basal ganglia output during rest and walk in a hemiparkinsonian rat. Exp. Neurol. 221, 307–319 10.1016/j.expneurol.2009.11.016 19948166 PMC3384738

[B5] Bereshpolova Y. Stoelzel C. R. Zhuang J. Amitai Y. Alonso J. M. Swadlow H. A. (2011). Getting drowsy? Alert/nonalert transitions and visual thalamocortical network dynamics. J. Neurosci. 31, 17480–17487 10.1523/JNEUROSCI.2262-11.2011 22131409 PMC6623815

[B6] Bodor A. L. Giber K. Rovo Z. Ulbert I. Acsady L. (2008). Structural correlates of efficient GABAergic transmission in the basal ganglia-thalamus pathway. J. Neurosci. 28, 3090–3102 10.1523/JNEUROSCI.5266-07.2008 18354012 PMC2670451

[B7] Boyden E. S. Zhang F. Bamberg E. Nagel G. Deisseroth K. (2005). Millisecond-timescale, genetically targeted optical control of neural activity. Nat. Neurosci. 8, 1263–1268 10.1038/nn1525 16116447

[B8] Brazhnik E. Cruz A. V. Avila I. Wahba M. I. Novikov N. Ilieva N. M. (2012). State-dependent spike and local field synchronization between motor cortex and substantia nigra in hemiparkinsonian rats. J. Neurosci. 32, 7869–7880 10.1523/JNEUROSCI.0943-12.2012 22674263 PMC3423905

[B9] Browne S. H. Kang J. Akk G. Chiang L. W. Schulman H. Huguenard J. R. (2001). Kinetic and pharmacological properties of GABA(A) receptors in single thalamic neurons and GABA(A) subunit expression. J. Neurophysiol. 86, 2312–2322 11698521 10.1152/jn.2001.86.5.2312

[B10] Cox C. L. Huguenard J. R. Prince D. A. (1997). Nucleus reticularis neurons mediate diverse inhibitory effects in thalamus. Proc. Natl. Acad. Sci. U.S.A. 94, 8854–8859 10.1073/pnas.94.16.8854 9238067 PMC23165

[B11] Delong M. R. (1971). Activity of pallidal neurons during movement. J. Neurophysiol. 34, 414–427 4997823 10.1152/jn.1971.34.3.414

[B13] Delong M. R. (1990). Primate models of movement disorders of basal ganglia origin. Trends Neurosci. 13, 281–285 10.1016/0166-2236(90)90110-V 1695404

[B14] Franklin K. B. J. Paxinos G. (2008). The Mouse Brain in Stereotaxic Coordinates. Elsevier: Academic Press

[B15] Fritschy J. M. Paysan J. Enna A. Mohler H. (1994). Switch in the expression of rat gaba(a)-receptor subtypes during postnatal-development—an immunohistochemical study. J. Neurosci. 14, 5302–5324 8083738 10.1523/JNEUROSCI.14-09-05302.1994 PMC6577100

[B16] Georgopoulos A. P. Delong M. R. Crutcher M. D. (1983). Relations between parameters of step-tracking movements and single cell discharge in the globus pallidus and subthalamic nucleus of the behaving monkey. J. Neurosci. 3, 1586–1598 6875658 10.1523/JNEUROSCI.03-08-01586.1983 PMC6564524

[B17] Goldberg J. A. Boraud T. Maraton S. Haber S. N. Vaadia E. Bergman H. (2002). Enhanced synchrony among primary motor cortex neurons in the 1-methyl-4-phenyl-1,2,3,6-tetrahydropyridine primate model of Parkinson's disease. J. Neurosci. 22, 4639–4653 12040070 10.1523/JNEUROSCI.22-11-04639.2002 PMC6758785

[B18] Goldberg J. H. Farries M. A. Fee M. S. (2012). Integration of cortical and pallidal inputs in the basal ganglia-recipient thalamus of singing birds. J. Neurophysiol. 108, 1403–1429 10.1152/jn.00056.2012 22673333 PMC3544964

[B19] Graybiel A. M. (2005). The basal ganglia: learning new tricks and loving it. Curr. Opin. Neurobiol. 15, 638–644 10.1016/j.conb.2005.10.006 16271465

[B20] Gunaydin L. A. Yizhar O. Berndt A. Sohal V. S. Deisseroth K. Hegemann P. (2010). Ultrafast optogenetic control. Nat. Neurosci. 13, 387–392 10.1038/nn.2495 20081849

[B21] Gurney K. Prescott T. J. Redgrave P. (2001). A computational model of action selection in the basal ganglia. I. A new functional anatomy. Biol. Cybern. 84, 401–410 10.1007/PL00007984 11417052

[B22] Herd M. B. Brown A. R. Lambert J. J. Belelli D. (2013). Extrasynaptic GABA(A) receptors couple presynaptic activity to postsynaptic inhibition in the somatosensory thalamus. J. Neurosci. 33, 14850–14868 10.1523/JNEUROSCI.1174-13.2013 24027285 PMC6705167

[B23] Hodaie M. Cordella R. Lozano A. M. Wennberg R. Dostrovsky J. O. (2006). Bursting activity of neurons in the human anterior thalamic nucleus. Brain Res. 1115, 1–8 10.1016/j.brainres.2006.07.085 16962566

[B24] Humphries M. D. Stewart R. D. Gurney K. N. (2006). A physiologically plausible model of action selection and oscillatory activity in the basal ganglia. J. Neurosci. 26, 12921–12942 10.1523/JNEUROSCI.3486-06.2006 17167083 PMC6674973

[B25] Huntsman M. M. Huguenard J. R. (2000). Nucleus-specific differences in GABA(A)-receptor-mediated inhibition are enhanced during thalamic development. J. Neurophysiol. 83, 350–358 10634878 10.1152/jn.2000.83.1.350

[B26] Jahnsen H. Llinas R. (1984a). Electrophysiological properties of guinea-pig thalamic neurons - an *invitro* study. J. Physiol. (Lond.) 349, 205–226 6737292 10.1113/jphysiol.1984.sp015153 PMC1199334

[B27] Jahnsen H. Llinas R. (1984b). Ionic basis for the electroresponsiveness and oscillatory properties of guinea-pig thalamic neurons *invitro*. J. Physiol. (Lond.) 349, 227–247 6737293 10.1113/jphysiol.1984.sp015154 PMC1199335

[B28] Kultas-Ilinsky K. Sivan-Loukianova E. Ilinsky I. A. (2003). Reevaluation of the primary motor cortex connections with the thalamus in primates. J. Comp. Neurol. 457, 133–158 10.1002/cne.10539 12541315

[B29] Kuramoto E. Fujiyama F. Nakamura K. C. Tanaka Y. Hioki H. Kaneko T. (2011). Complementary distribution of glutamatergic cerebellar and GABAergic basal ganglia afferents to the rat motor thalamic nuclei. Eur. J. Neurosci. 33, 95–109 10.1111/j.1460-9568.2010.07481.x 21073550

[B30] Kuramoto E. Furuta T. Nakamura K. C. Unzai T. Hioki H. Kaneko T. (2009). Two types of thalamocortical projections from the motor thalamic nuclei of the rat: a single neuron-tracing study using viral vectors. Cereb. Cortex 19, 2065–2077 10.1093/cercor/bhn231 19174446

[B31] Laudes T. Meis S. Munsch T. Lessmann V. (2012). Impaired transmission at corticothalamic excitatory inputs and intrathalamic gabaergic synapses in the ventrobasal thalamus of heterozygous bdnf knockout mice. Neuroscience 222, 215–227 10.1016/j.neuroscience.2012.07.005 22796079

[B32] Leblois A. Bodor A. L. Person A. L. Perkel D. J. (2009). Millisecond timescale disinhibition mediates fast information transmission through an avian basal ganglia loop. J. Neurosci. 29, 15420–15433 10.1523/JNEUROSCI.3060-09.2009 20007467 PMC2819911

[B33] Llinas R. Jahnsen H. (1982). Electrophysiology of mammalian thalamic neurons *in vitro*. Nature 297, 406–408 10.1038/297406a0 7078650

[B34] Llinás R. R. Steriade M. (2006). Bursting of thalamic neurons and states of vigilance. J. Neurophysiol. 95, 3297–3308 10.1152/jn.00166.2006 16554502

[B35] Luo M. M. Perkel D. J. (1999a). A GABAergic, strongly inhibitory projection to a thalamic nucleus in the zebra finch song system. J. Neurosci. 19, 6700–6711 10414999 10.1523/JNEUROSCI.19-15-06700.1999 PMC6782801

[B36] Luo M. M. Perkel D. J. (1999b). Long-range GABAergic projection in a circuit essential for vocal learning. J. Comp. Neurol. 403, 68–84 10.1002/(SICI)1096-9861(19990105)403:1<68::AID-CNE6>3.0.CO;2-5 10075444

[B37] Luo M. M. Perkel D. J. (2002). Intrinsic and synaptic properties of neurons in an avian thalamic nucleus during song learning. J. Neurophysiol. 88, 1903–1914 12364516 10.1152/jn.2002.88.4.1903

[B38] Macia F. Escola L. Guehl D. Michelet T. Bioulac B. Burbaud P. (2002). Neuronal activity in the monkey motor thalamus during bicuculline-induced dystonia. Eur. J. Neurosci. 15, 1353–1362 10.1046/j.1460-9568.2002.01964.x 11994129

[B39] Magnin M. Morel A. Jeanmonod D. (2000). Single-unit analysis of the pallidum, thalamus and subthalamic nucleus in parkinsonian patients. Neuroscience 96, 549–564 10.1016/S0306-4522(99)00583-7 10717435

[B40] Murer M. G. Riquelme L. A. Tseng K. Y. Pazo J. H. (1997). Substantia nigra pars reticulata single unit activity in normal and 60HDA-lesioned rats: Effects of intrastriatal apomorphine and subthalamic lesions. Synapse 27, 278–293 10.1002/(SICI)1098-2396(199712)27:4<278::AID-SYN2>3.0.CO;2-9 9372551

[B41] Nagel G. Szellas T. Huhn W. Kateriya S. Adeishvili N. Berthold P. (2003). Channelrhodopsin-2, a directly light-gated cation-selective membrane channel. Proc. Natl. Acad. Sci. U.S.A. 100, 13940–13945 10.1073/pnas.1936192100 14615590 PMC283525

[B42] Nakamura K. C. Sharott A. Magill P. J. (2014). Temporal coupling with cortex distinguishes spontaneous neuronal activities in identified basal ganglia-recipient and cerebellar-recipient zones of the motor thalamus. Cereb. Cortex 24, 81–97 10.1093/cercor/bhs287 23042738 PMC3862266

[B43] Nambu A. (2008). Seven problems on the basal ganglia. Curr. Opin. Neurobiol. 18, 595–604 10.1016/j.conb.2008.11.001 19081243

[B44] Pare D. Dossi R. C. Steriade M. (1990). Neuronal basis of the parkinsonian resting tremor - a hypothesis and its implications for treatment. Neuroscience 35, 217–226 10.1016/0306-4522(90)90077-H 2199839

[B45] Peden D. R. Petitjean C. M. Herd M. B. Durakoglugil M. S. Rosahl T. W. Wafford K. (2008). Developmental maturation of synaptic and extrasynaptic GABA(A) receptors in mouse thalamic ventrobasal neurones. J. Physiol. (Lond.) 586, 965–987 10.1113/jphysiol.2007.145375 18063661 PMC2375643

[B46] Person A. L. Perkel D. J. (2005). Unitary IPSPs drive precise thalamic spiking in a circuit required for learning. Neuron 46, 129–140 10.1016/j.neuron.2004.12.057 15820699

[B47] Person A. L. Perkel D. J. (2007). Pallidal neuron activity increases during sensory relay through thalamus in a songbird circuit essential for learning. J. Neurosci. 27, 8687–8698 10.1523/JNEUROSCI.2045-07.2007 17687046 PMC6672941

[B48] Ramcharan E. J. Gnadt J. W. Sherman S. M. (2000). Burst and tonic firing in thalamic cells of unanesthetized, behaving monkeys. Vis. Neurosci. 17, 55–62 10.1017/S0952523800171056 10750826

[B49] Raz A. Vaadia E. Bergman H. (2000). Firing patterns and correlations of spontaneous discharge of pallidal neurons in the normal and the tremulous 1-methyl-4-phenyl-1,2,3,6-tetrahydropyridine vervet model of parkinsonism. J. Neurosci. 20, 8559–8571 11069964 10.1523/JNEUROSCI.20-22-08559.2000 PMC6773163

[B50] Ruskin D. N. Bergstrom D. A. Kaneoke Y. Patel B. N. Twery M. J. Walters J. R. (1999). Multisecond oscillations in firing rate in the basal ganglia: Robust modulation by dopamine receptor activation and anesthesia. J. Neurophysiol. 81, 2046–2055 10322046 10.1152/jn.1999.81.5.2046

[B51] Sakai S. T. Grofova I. Bruce K. (1998). Nigrothalamic projections and nigrothalamocortical pathway to the medial agranular cortex in the rat: Single- and double-labeling light and electron microscopic studies. J. Comp. Neurol. 391, 506–525 10.1002/(SICI)1096-9861(19980222)391:4<506::AID-CNE7>3.0.CO;2-4 9486828

[B52] Sherman S. M. (2001). Tonic and burst firing: dual modes of thalamocortical relay. Trends Neurosci. 24, 122–126 10.1016/S0166-2236(00)01714-8 11164943

[B53] Sherman S. M. Guillery R. W. (2002). The role of the thalamus in the flow of information to the cortex. Philos. Trans. R. Soc. B Biol. Sci. 357, 1695–1708 10.1098/rstb.2002.1161 12626004 PMC1693087

[B54] Stoelzel C. R. Bereshpolova Y. Swadlow H. A. (2009). Stability of thalamocortical synaptic transmission across awake brain states. J. Neurosci. 29, 6851–6859 10.1523/JNEUROSCI.5983-08.2009 19474312 PMC2713605

[B56] Vitek J. L. Ashe J. Delong M. R. Alexander G. E. (1994). Physiological-properties and somatotopic organization of the primate motor thalamus. J. Neurophysiol. 71, 1498–1513 8035231 10.1152/jn.1994.71.4.1498

[B57] Walter J. T. Khodakhah K. (2006). The linear computational algorithm of cerebellar Purkinje cells. J. Neurosci. 26, 12861–12872 10.1523/JNEUROSCI.4507-05.2006 17167077 PMC6674952

[B58] Walter J. T. Khodakhah K. (2009). The advantages of linear information processing for cerebellar computation. Proc. Natl. Acad. Sci. U.S.A. 106, 4471–4476 10.1073/pnas.0812348106 19234116 PMC2657437

[B59] Wei H. Bonjean M. Petry H. M. Sejnowski T. J. Bickford M. E. (2011). Thalamic burst firing propensity: a comparison of the dorsal lateral geniculate and pulvinar nuclei in the tree shrew. J. Neurosci. 31, 17287–17299 10.1523/JNEUROSCI.6431-10.2011 22114295 PMC3236686

[B60] Wichmann T. Bergman H. Starr P. A. Subramanian T. Watts R. L. Delong M. R. (1999). Comparison of MPTP-induced changes in spontaneous neuronal discharge in the internal pallidal segment and in the substantia nigra pars reticulata in primates. Exp. Brain Res. 125, 397–409 10.1007/s002210050696 10323285

[B61] Wichmann T. Delong M. R. (1996). Functional and pathophysiological models of the basal ganglia. Curr. Opin. Neurobiol. 6, 751–758 10.1016/S0959-4388(96)80024-9 9000030

[B62] Wichmann T. Dostrovsky J. O. (2011). Pathological basal ganglia activity in movement disorders. Neuroscience 198, 232–244 10.1016/j.neuroscience.2011.06.048 21723919 PMC3209553

[B63] Wichmann T. Kliem M. A. (2004). Neuronal activity in the primate substantia nigra pars reticulata during the performance of simple and memory-guided elbow movements. J. Neurophysiol. 91, 815–827 10.1152/jn.01180.2002 14762150

[B64] Zhang S. L. J. Huguenard J. R. Prince D. A. (1997). GABA(A) receptor-mediated Cl- currents in rat thalamic reticular and relay neurons. J. Neurophysiol. 78, 2280–2286 9356381 10.1152/jn.1997.78.5.2280

[B65] Zhang Z. W. Zak J. D. Liu H. (2010). MeCP2 is required for normal development of gabaergic circuits in the thalamus. J. Neurophysiol. 103, 2470–2481 10.1152/jn.00601.2009 20200124 PMC2867574

