# Supplementary material for: Optogenetic activation of nigral inhibitory inputs to motor thalamus in the mouse reveals classic inhibition with little potential for rebound activation
Source: Front Cell Neurosci. 2014 Feb 11;8:36. doi: 10.3389/fncel.2014.00036 (PMC3920182; doi:10.3389/fncel.2014.00036)

Two recordings, in which stimulation was performed at 3 different light intensities by inserting neutral density filters into the light path of a 100W mercury light source. Averaged IPSCs for a 1 ms duration full field light flash presented through our 60x objective are shown.
Note the increased response delay at lower stimulus intensity, suggesting a delayed initiation of action.
Also note the appearance of multiple peaks for higher stimulation intensities. We can not differentiate between multiple fibers being activated at different delays or a single fiber firing a burst. However, for a 1 ms stimulus a burst response in a nigral fiber may appear somewhat unlikely.

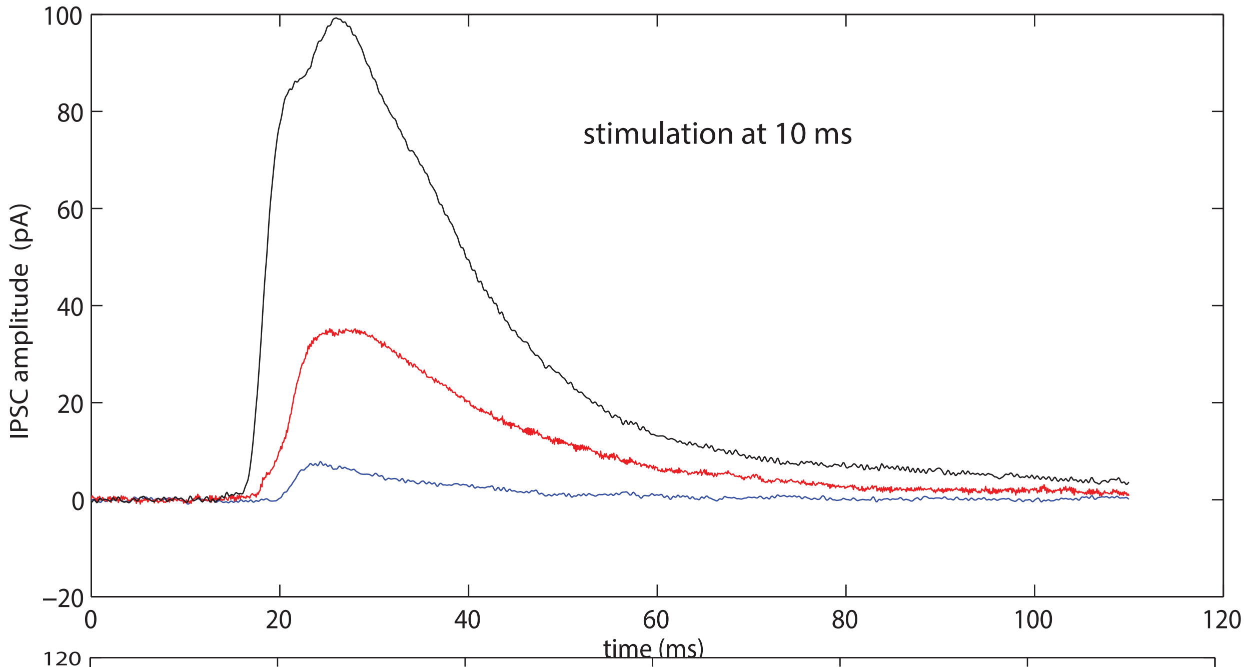

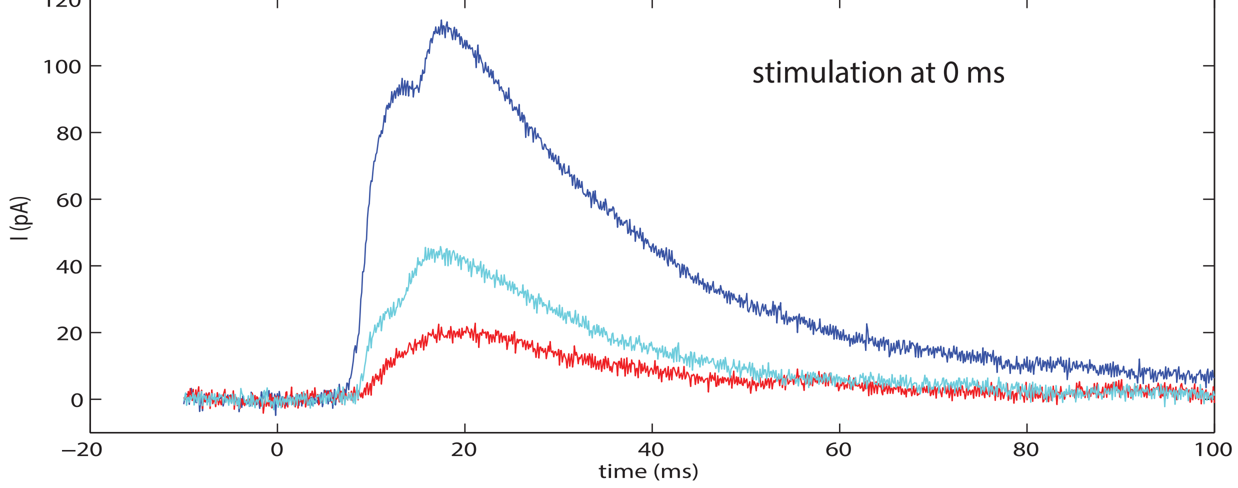

A third recording, stimulated at 2 light intensities. Single IPSCs on the left, average on the right. Stimulation at 0 ms.

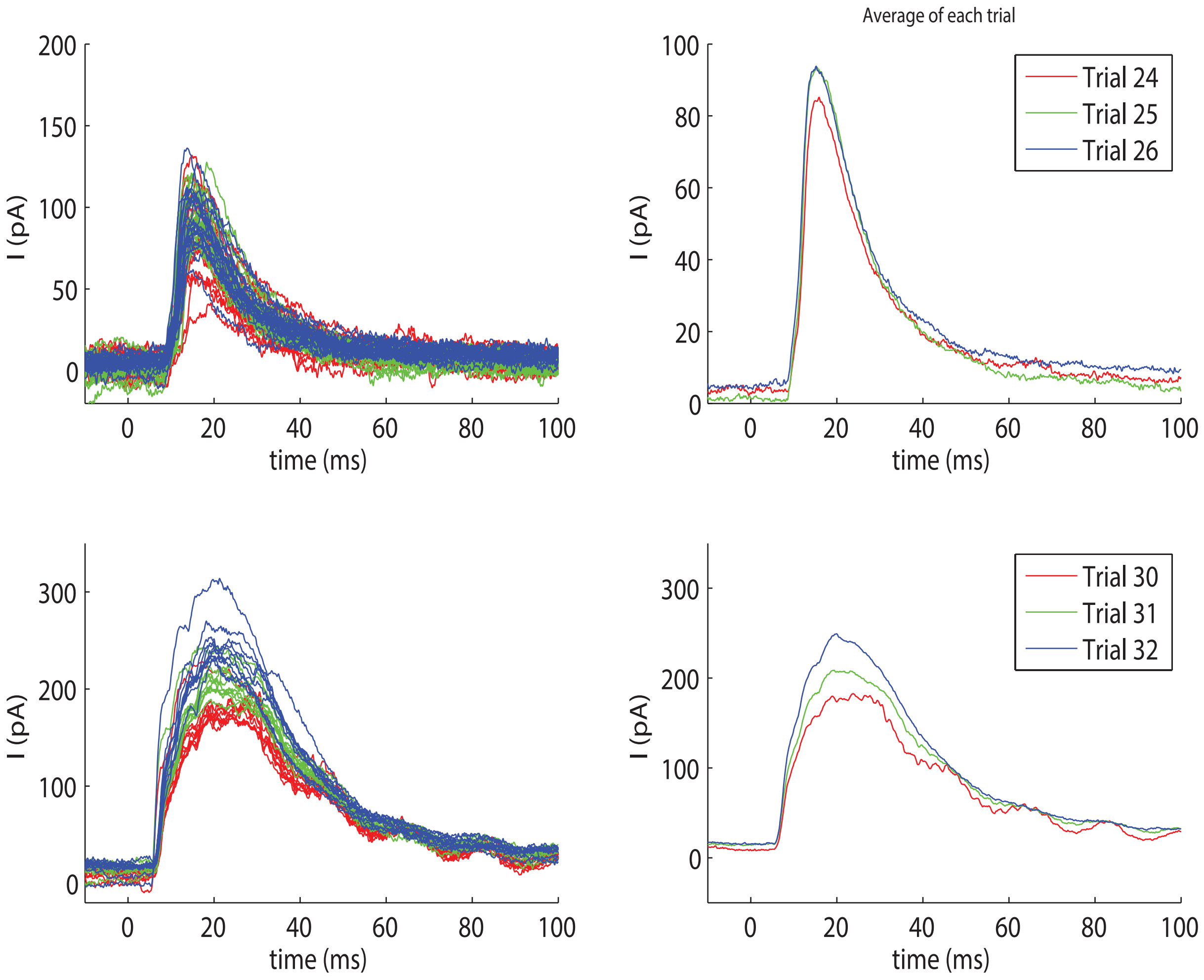

Supplement: Supplementary file 1 [file DataSheet1.PDF]
